# Thermoluminescence of coral skeletons: a high-sensitivity proxy of diagenetic alteration of aragonite

**DOI:** 10.1038/s41598-017-18269-y

**Published:** 2017-12-21

**Authors:** Noriyuki Takada, Atsushi Suzuki, Hiroshi Ishii, Katsuyuki Hironaka, Takayuki Hironiwa

**Affiliations:** 1 Research Institute for Sustainable Chemistry, National Institute of Advanced Industrial Science and Technology, Central 5, 1-1-1 Higashi, Tsukuba, Ibaraki 305-8565 Japan; 2 Geological Survey of Japan, National Institute of Advanced Industrial Science and Technology, Central 7, 1-1-1 Higashi, Tsukuba, Ibaraki 305-8567 Japan; 3 Ueshima Seisakusho Co. Ltd., 6-5-22 Yaho, Kunitachi, Tokyo 186-0011 Japan; 4 Koga Isotope Ltd., 53-6 Jinbo, Koka, Shiga 520-3404 Japan

## Abstract

Diagenetic alteration of aragonite coral skeletons causes changes in their chemical and isotopic compositions. Such altered coral samples are unsuitable for age dating or paleoclimate reconstructions. Recently developed microanalysis techniques have elucidated secondary aragonite precipitation and calcite overgrowth on primary aragonitic coral skeletons, but an effective screening method for bulk samples is still desirable. Although powder X-ray diffraction (XRD) analysis is widely used for this purpose, its detection limit for calcite (1–2% at best) is not sufficient to detect very small amounts of diagenetic calcite. Here, we propose that thermoluminescence (TL) spectra can be used to detect the presence of tiny amounts of secondary calcite in coral skeletons. We used a TL spectrometer with a Fourier-transform detector to detect the calcite component in TL spectra of powdered skeletons of modern and fossil corals (from 127 ka and 3.5 Ma) in which calcite was not detectable by XRD. The key element is manganese, because the TL emission efficiency and the partition coefficient of Mn are greater for calcite than for aragonite. As a result, the calcite spectral component becomes evident. Thus, the TL spectroscopic technique is a highly sensitive tool for screening fossil corals for diagenetic alteration.

## Introduction

Geological samples such as coral skeletons^1,2^ and foraminifer tests that have been altered by diagenesis over very long periods lose their original paleoenvironmental information. Therefore, screening for pristine samples, ones that have not experienced diagenetic alteration, is important for the extraction of accurate geological information. In corals, diagenetic alterations may include dissolution of primary coral aragonite, infilling of skeletal pore spaces with secondary cements, and recrystallization of coral aragonite to calcite^2^. In this study, we focused on post-depositional changes to skeletons originally composed of aragonite (orthorhombic CaCO_3_) whereby the aragonite is recrystallized to stable calcite (rhombohedral CaCO_3_) in a subaerial setting under normal temperature and pressure conditions, in contrast to secondary aragonite, which forms during diagenesis in a submarine environment^3–5^.

Recently developed microanalysis techniques such as secondary ion mass spectrometry and laser-ablation induced coupled plasma mass spectrometry have contributed to the elucidation of micro-scale features such as secondary aragonite precipitation and calcite overgrowth on the primary coral skeleton^2^ (Supplementary information 1), but an effective screening method for bulk samples is still desirable because pristine bulk samples (milligram-scale powdered samples from millimetre-scale specimens) are required for age dating and geochemical measurements. Powder X-ray diffraction (XRD) analysis has been widely used to discern traces of diagenetic alteration in bulk samples, but its detection limit (1–2% at best)^6,7^ is inadequate for use as a precise screening method for pristine coral samples. For example, the presence of less than 1% secondary calcite can cause estimated temperature deviations of up to ~1 °C^8^. Thus, for reconstructing paleo-El Niño events in tropical regions, for instance, detection of subpercentage levels of secondary calcite is crucial because El Niño events are defined by a temperature anomaly in the Niño 3.4 region of more than 0.5 °C^9^. Trace elements in carbonate are important tracers of diagenetic alteration^10^, and the contents of manganese, iron, and zinc progressively increase in altered carbonate^11^. In particular, the partition coefficient of manganese in calcite is greater than unity, whereas in aragonite it is less than unity. Thus, in a closed aragonite/calcite transformation system, secondary calcite is greatly enriched in manganese relative to the parent aragonite as a result of dissolution and reprecipitation^10^. For example, an increase in the Mn/Ca ratio in earlier precipitated parts of a massive, 400-year-old *Porites* coral from the Galapagos Islands was hypothesized to be due to an increase in diagenetically emplaced Mn oxide or other phases^12^. Manganese is incorporated into the crystal lattice of coral aragonite, where it typically occurs at a concentration of 8–150 ppb (Mn/Ca ratio, 15–280 nmol/mol)^12^, and manganese in coral skeletons, because of its redox-sensitive behaviour in aquatic environments, has recently been used as a proxy for river discharge or biological activity^13,14^. Although inductively coupled plasma-mass spectrometry (ICP-MS) has been widely used for precise determination of trace element concentrations in coral skeletons, a reliable trace-element-based proxy for diagenetic alteration has not yet been discovered. Here we propose that luminescence technologies, such as thermoluminescence (TL), cathodoluminescence (CL), and photoluminescence (PL), may provide a simple alternative screening method for coral diagenesis.

Minerals such as calcite have been observed by TL^15,16^ and CL spectroscopy^17,18^ for many years. The luminescence of minerals is attributed to the presence of manganese ion (Mn^2+^) impurities embedded within them that act as recombination sites, and the emission transition of Mn^2+^ depends on the crystal structure of the mineral^19,20^. According to crystal field theory^21,22^, the emission transition of Mn^2+^ should occur at a higher energy in an aragonite crystal than in a calcite crystal because of the larger average metal-to-oxygen distance in aragonite. Consequently, it should be possible to separate the emission spectrum of calcite from that of aragonite, which in turn suggests that it might be possible to use luminescence technologies to evaluate diagenetic alteration of aragonite.

The reported TL intensity of aragonite is considerably weaker than that of calcite^23^. Therefore, in this study we decided to use a multichannel Fourier-transform spectrometer (FTS), which can detect very weak TL signals^24^. Unlike a grating dispersive spectrometer, which has small optical throughput because an entrance slit is necessary to resolve the spectrum, a FTS can realize a large optical throughput because it uses an interferometer and no slit is necessary. Thus, even very weak luminescence can be compensated by the total intensity radiated from a two-dimensional, spread-out sample.

In this study, we used a FTS to acquire TL spectra of modern and fossil corals and then conducted PL, electron spin resonance (ESR), and powder XRD analyses to confirm the existence of a calcite domain generated by diagenetic alteration in the samples. We also examined why it is possible to detect TL emissions even from the very small amounts of calcite in the corals.

## Materials and Methods

We examined modern and fossil skeletal samples from massive *Porites* spp. coral colonies. Modern coral samples were collected by drilling on 14 January 1999 from the shaded side of a *Porites* colony at 5 m depth on the seaward side of the Pandora Reef, which is part of the Great Barrier Reef in Australia^25^. We examined fossil corals of two different ages. One fossil coral sample was collected at 3 m above the present mean sea level from an uplifted Pleistocene coral terrace on Yonaguni Island, part of the Southern Ryukyu Island chain (Japan); this sample (hereafter, the 127 ka coral) was previously determined by α-counting to have a U-Th age of 127 ± 6 ka^26^. The other fossil coral sample was collected from an exceptionally well preserved fossil coral from the Tartaro Formation on the island of Luzon in the Philippines^27^, where well-preserved specimens are found buried deep within layers of muddy sand. This age of this fossil coral (hereafter, the 3.5 Ma coral) was estimated to be approximately 3.5–3.8 million years by observations of associated nannofossil assemblages^27^.

Both the modern and fossil coral samples were cleaned by ultrasonic washing with Milli-Q^®^ water. Then samples were extracted for TL measurement using a procedure similar to that described by Gagan *et al*.^28^. A milling machine with a moveable table and a 2-mm-diameter drill bit was used for shaving off skeletal samples. The powdered samples fell onto a weighing paper placed beneath the coral slab mounted on the milling table. Several tens of milligrams of coral powder were collected from each sample for the TL measurements.

The TL spectra of the coral skeleton samples were detected by using a multichannel FTS (MS-8310, Ueshima Seisakusho Co. Ltd.), which consists of a sample holder with a heating unit, an optical system (polarizer/Savart-plate/polarizer/collecting-lens), and a Peltier-cooled charge-coupled device (CCD) as an image sensor. The observed interferogram was converted to a TL spectrum by fast Fourier transformation. This FTS system has the merits of a large throughput, simultaneous acquisition of the spectrum over a wide wavelength range (350–900 nm), and a high signal-to-noise ratio. However, the system has the disadvantage that its wavelength resolution is only approximately 10–20 nm.

TL occurs when materials are heated after being irradiated with X-rays, γ-rays, or UV-light^18,29,30^. Charge carriers generated by such irradiation are trapped in localized sites; the subsequent heating causes detrapping of the electrons, and TL is observed when the carriers recombine radiatively. TL intensity typically increases with the radiation exposure dose^31^. Thus, in this study, the coral skeletons were irradiated with ^60^Co γ-rays for a total dose of 10 kGy at room temperature in the atmosphere before the TL spectra were measured by FTS. Detection of emission signals began within about 5 s after the irradiated coral was introduced into the sample holder, which had been preheated to 200 °C. The TL spectrum was evaluated by using the interferogram signal accumulated over 1 min.

PL spectra of the coral samples were measured with an optical microspectroscopy system (Nikon Eclipse E600, UV-1A filter cube (Ex 365/10, DM 400, BA400), ×10 objective lens) equipped with an optical-fibre-connected polychrometer CCD detector (Acton SpectraPro 2150, Princeton Instruments PIXIS 1024) at room temperature under normal air conditions. The excitation wavelength was fixed to the 365-nm bright line of a mercury lamp.

CL spectra of the 3.5 Ma fossil coral were acquired with a Schottky Field Emission Scanning Electron Microscope (JEOL JSM-7100F/TTLS) equipped with an optical-fibre-connected polychrometer CCD detector (HORIBA iHR-320, HORIBA Jobin Yvon Synapse CCD BIUV) at room temperature. An electron beam energy of 5 kV was used for the CL spectroscopy.

For bulk Mn measurements, 5 mg of skeletal powder was dissolved in 5 ml of 2% HNO_3_ solution. The Mn concentrations in the skeletal samples were measured by ICP-MS (7700x ICP-MS, Agilent Technologies). The relative standard deviation for Mn was 3.4%. Concentrations of Mn in all bulk samples were measured in triplicate.

Powder particle size distributions were determined by laser diffraction (Malvern Mastersizer) following dispersion of dried samples in distilled water; the results are shown in Supplementary information 2.

Powder XRD analysis of the coral skeleton powder samples was performed by using an X-ray powder diffractometer (Rigaku Ultima IV Protectus) with Cu*k*α radiation (40 kV and 40 mA) and an Ni filter with a scanning speed of 0.5° 2θ/min.

ESR spectra were detected at room temperature under normal air conditions with a JEOL X-band JES-FR30EX spectrometer (microwave power 0.4 mW; microwave frequency 9.42 GHz; sweep width ± 7.5 mT; modulation amplitude 0.032 mT).

## Results and Discussion

**Figure 1:**
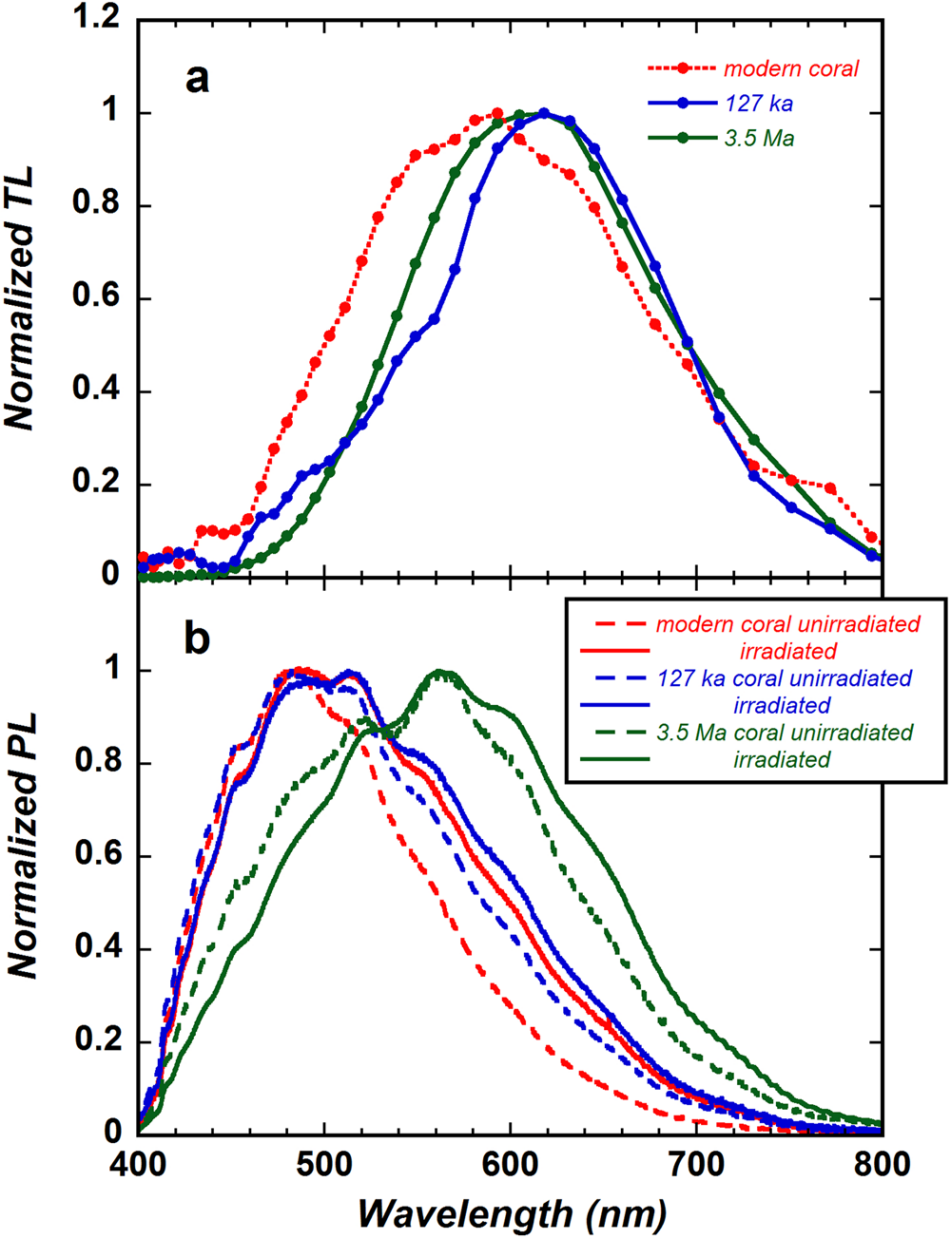
(**a**) Normalized thermoluminescence (TL) spectra of samples of modern coral (Pandora Reef, Great Barrier Reef), 127 ka coral (Pleistocene coral from Yonaguni Island, Japan), and 3.5 Ma coral (Pliocene coral from Luzon Island, Philippines). (**b**) Normalized photoluminescence (PL) spectra of unirradiated (broken lines) and γ-ray irradiated (solid line) corals.

The normalized TL spectrum of the modern coral was characterized by a broad emission band centred near 590 nm, whereas the central wavelength of the normalized TL spectra of both the 127 ka and 3.5 Ma fossil corals was about 620 nm (Fig. 1a). Medlin^15^ measured the broad TL spectra of synthetic aragonite samples at low temperatures (180 K and 250 K), and Khanlary *et al*.^32^ observed the TL spectra of aragonite at temperatures higher than about 200 °C. The TL of aragonite is known to be attributable to the presence of Mn^2+^ impurities, which act as recombination sites and emission centres^19^. The Mn concentrations determined by our ICP-MS analysis of the modern, 127 ka, and 3.5 Ma corals were 0.15, 0.12, and 2.0 ppm, respectively, whereas Medlin^23^ and Calderon *et al*.^19^ reported much higher Mn concentrations of >10 ppm in their aragonite samples. Therefore, to detect the TL emissions, which we expected to be weak owing to the low Mn concentrations in the coral skeletons, we subjected the coral samples to a large radiation exposure dose (10 kGy), which increases TL intensity, and we utilized a highly sensitive FTS system and long signal accumulation times (1 min).

When Mn is present as isolated impurities with low concentrations, clearly resolved emission bands consisting of a series of Mn-transition lines have been reported^32–34^. However, instead of the expected narrow emission bands, we obtained broad emission bands (Fig. 1a). The reason for the broad bands is unclear, but they may be caused by the superposition or broadening of emission transitions due to different sample states (Mn^2+^ distribution, crystal structure, impurities, defects, etc.). Micro-scale observations using spectroscopy techniques such as CL or Raman spectroscopy should be performed to examine the sample state^35–37^. However, we believe that the low wavelength resolution of the FTS system did not have much impact on the results of this study.

In the modern, 127 ka, and 3.5 Ma corals, compared with spectra acquired before γ-ray irradiation, the PL spectra were shifted to the low-energy side after γ-ray irradiation (Fig. 1b). However, the shift was larger for the modern coral than for the fossil corals. As a result, the PL spectra of the modern and 127 ka corals after γ-ray irradiation showed relatively small differences, although the difference in the PL spectrum between the 3.5 Ma coral and the other two corals remained large. Furthermore, the PL spectrum shows not only Mn-activated luminescence but also luminescence activated by lattice defects (intrinsic defects) in aragonite crystals^38^. We therefore examined the evidence for an increase in defect density and competitive emissions between Mn and defects.

**Figure 2:**
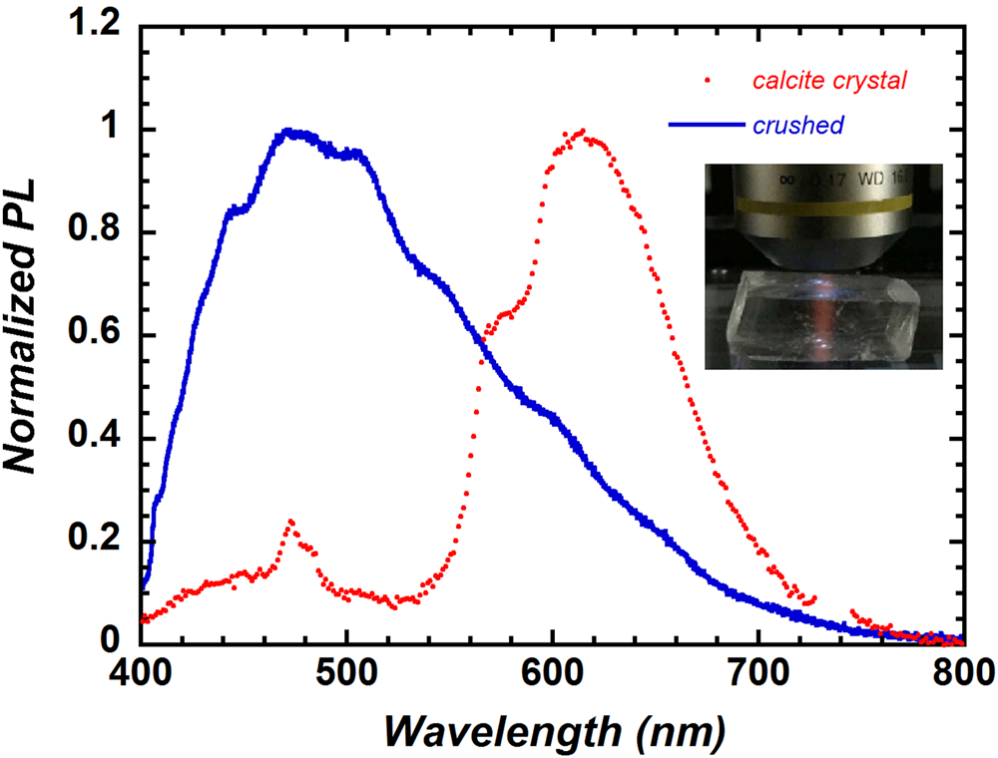
Normalized photoluminescence (PL) spectra of a whole calcite crystal and a crushed crystal. The main peak for the whole crystal is at 620 nm, whereas for the crushed crystal, there is instead a broad band centred at 480 nm, similar to the PL spectrum of unirradiated modern coral shown in Fig. 1b. The inset shows a red emission from a calcite crystal irradiated by UV light (365 nm) through the objective lens.

We first compared photoluminescence spectra between a single whole calcite crystal and the same crystal after it was mechanically crushed. A photograph of the whole calcite crystal (Fig. 2 inset) shows red PL derived from Mn^2+^ impurities in the crystal. When the calcite crystal was mechanically crushed, however, the red PL disappeared and a new blue emission appeared. The PL spectrum of the whole calcite crystal showed secondary peaks of Dy^3+^ near 475 and 580 nm in addition to the main peak of Mn^2+^ at 620 nm (Fig. 2). In contrast, the PL spectrum of the crushed crystal was centred near 480 nm and was similar to the PL spectra of the modern and 127 ka corals (Fig. 1b). This result suggests that intrinsic defects (lattice defects and dislocations) are formed by mechanical crushing.

To elucidate the relationship between intrinsic defects and PL spectra, we used an agate mortar and pestle to grind the modern coral powder for 5 min and then measured the PL spectrum of the ground powder without γ-ray irradiation. The PL spectrum acquired after grinding showed almost the same red shift as the one shown by the PL spectrum acquired after γ-ray irradiation of the modern coral (Supplementary information 3, Fig. S2). This result suggests that the defects generated by γ-ray irradiation are similar to those produced by mechanical grinding.

**Figure 3:**
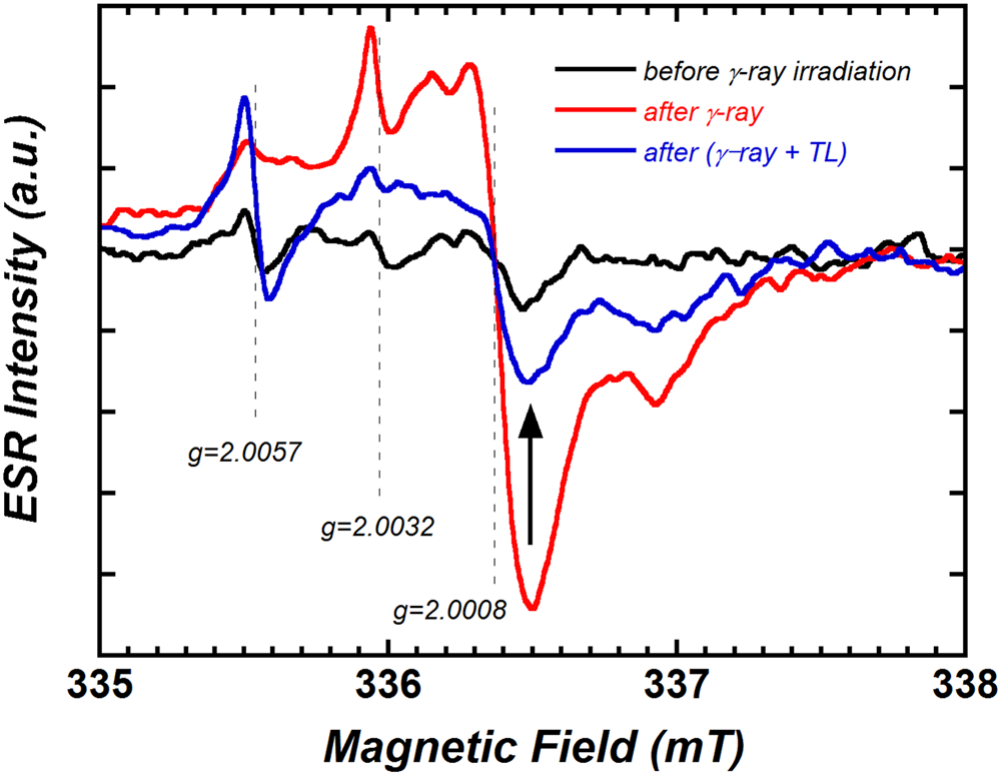
Electron spin resonance (ESR) spectra of the 127 ka coral before γ-ray irradiation, before normalized thermoluminescence (TL) measurement (with γ-ray irradiation), and after TL measurement (with γ-ray irradiation). The arrow indicates the remarkable change in the CO^2−^ radical (isotropic) signal from before to after TL measurement.

To obtain information on the nature of the intrinsic defects, we acquired ESR spectra of the corals before γ-ray irradiation, before TL measurement with γ-ray irradiation, and after TL measurement with γ-ray irradiation. The 127 ka coral can be presumed to have been subjected to long-term environmental irradiation, and its ESR spectrum without γ-ray irradiation displays clear signals at *g*-factors of *g* = 2.0008, 2.0032, and 2.0057 (Fig. 3), whereas the modern coral showed no such ESR signals. The signals at *g* = 2.0008, 2.0032, and 2.0057 are known to correspond to the CO_2_^−^ radical (isotropic)^39,40^, CO_3_^3−^ radical (axial)^41,42^, and SiO_2_^−^ radical (isotropic) paramagnetic centres^43,44^, respectively. The ESR spectrum of the γ-ray-irradiated 127 ka coral reveals a remarkable increase in the signals at *g* = 2.0008 and 2.0032. Furthermore, it is evident that these signals greatly decreased after TL measurement. Given that the ESR signals of the CO_2_^−^ and CO_3_^3−^ centres, which function as electron centres, decrease remarkably, the TL from the Mn^2+^ centre must be caused by the recombination of electrons thermally released from the CO_2_^−^ and CO_3_^3−^ electron centres with holes at Mn^3+^ sites^45^.

Comparison of the PL and TL spectra, with consideration of the ESR results, suggests that the red shift of the PL spectra after γ-ray irradiation, shown in Fig. 1b, is attributable to the formation of new defects or to an increase in the density of CO_2_^−^ and CO_3_^3−^ defects. An increase in defect density causes the extension of local lattice distortions, which in turn is expected to cause the spectral transition width (both PL and absorption) to become broader; thus, a red shift of the PL spectra might be observed. The similarity of the PL spectra of the modern and 127 ka corals after γ-ray irradiation suggests that the sites of the electron centre defects were the same between the modern and 127 ka corals. Additionally, the concentrations of Mn (i.e., emission centres) were also almost the same: 0.12 ppm for the 127 ka fossil coral and 0.15 ppm for the modern coral. Thus, it follows that the TL emission mechanism of the modern and 127 ka corals should be not significantly different. Despite the similarity of the TL mechanism between them, however, their TL spectra (Fig. 1a) were clearly different.

**Figure 4:**
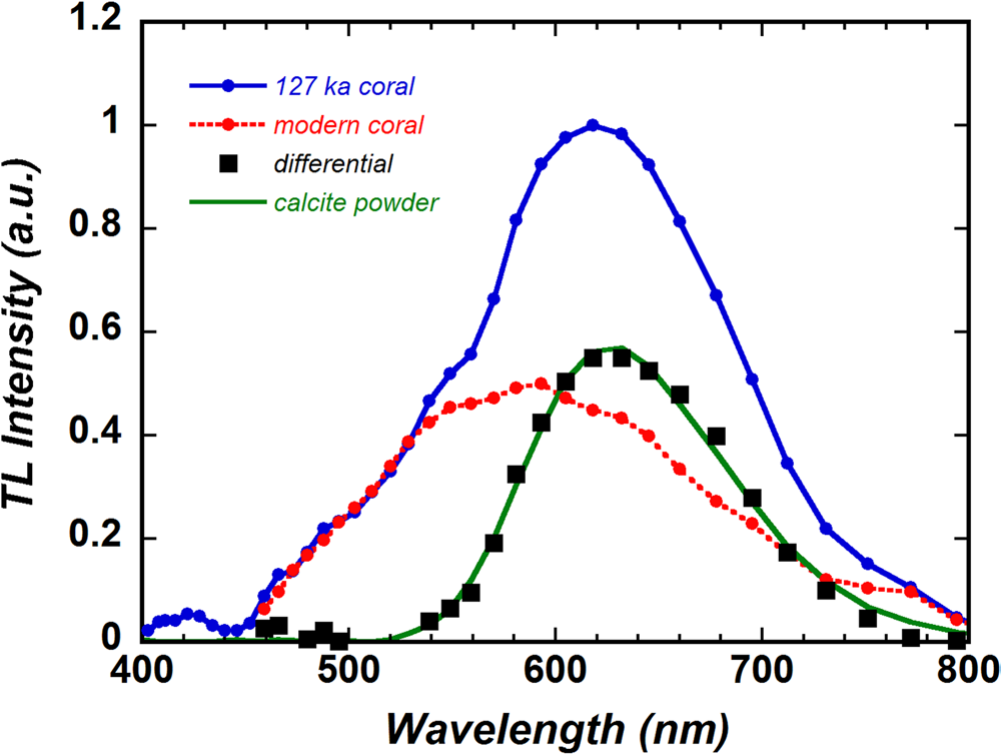
Normalized thermoluminescence (TL) spectrum of the 127 ka coral (same as in Fig. 1a) before subtraction of the TL spectrum of the aragonite modern coral and the differential data, shown by black squares. The TL spectrum of calcite powder reagent (99.95% calcite) is very similar to the differential data.

It is reasonable to hypothesize that the TL spectrum of the 127 ka coral contains the spectral component of the modern coral. Therefore, under the assumption that the shoulder on the short-wavelength side of the fossil coral spectrum accorded with that on the modern coral spectrum, we adjusted the TL spectrum of the modern coral before subtracting it from that of the fossil coral. The shoulders of the two spectra matched when the normalized TL peak intensity of the modern coral (Fig. 1a) reached about 0.5. Figure 4 shows the TL spectrum of the fossil coral (blue solid line with circles), the adjusted TL spectrum of the modern coral (red broken line with circles), and the differential data (squares). To elucidate the origin of the differential data, we performed TL measurements on high-purity calcite powder reagent (99.95%, Wako Pure Chemical Industries, Ltd.) (green solid line in Fig. 4). The differential data are obviously in good agreement with the TL spectrum of the calcite reagent, which suggests that the aragonite of the fossil coral probably includes a calcite component.

**Figure 5:**
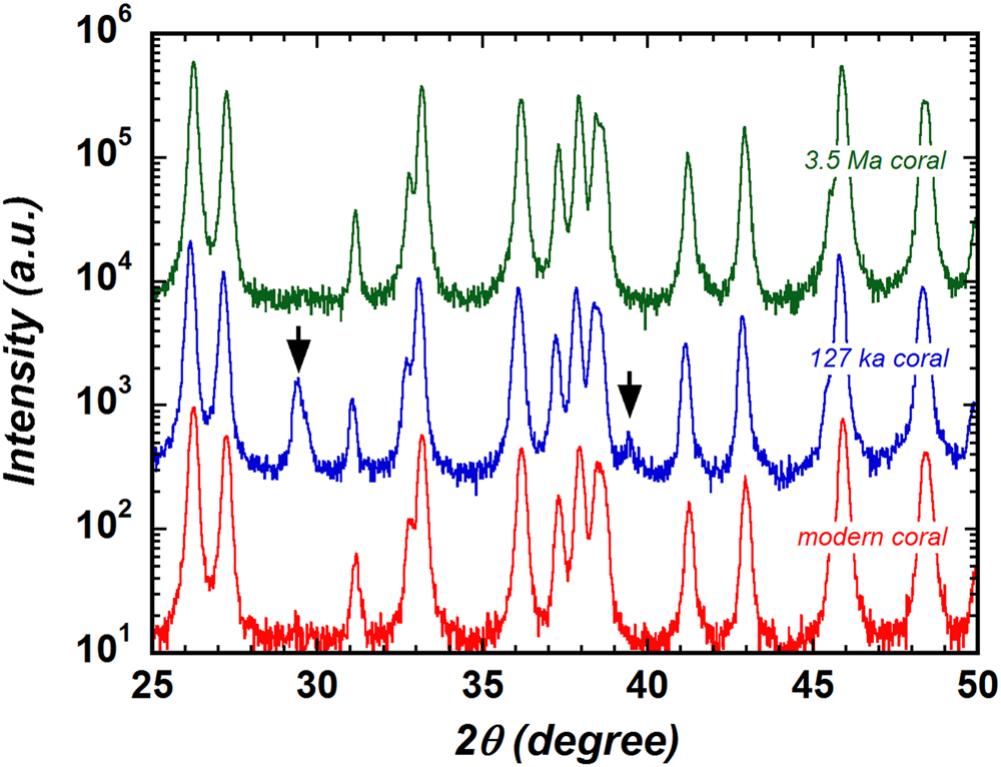
X-ray diffraction (XRD) spectra of the 3.5 Ma coral (upper green line), 127 ka coral (middle blue line), and modern coral (lower red line) powders. The baselines of these spectra have been shifted to facilitate comparison. The arrows indicate calcite peaks, which show that a calcite domain is present in the 127 ka coral.

The powder XRD spectra of the modern, 127 ka, and 3.5 Ma corals are shown in Fig. 5. The XRD spectrum of the 127 ka coral clearly shows diffraction lines for calcite (arrows). By using equation (9) of Kontoyannis and Vagenas^46^, we calculated the molar ratio of calcite to aragonite to be about 0.025. The presence of a calcite component in the 127 ka coral is an indication that diagenetic alteration of aragonite to calcite has occurred. Additionally, it is very interesting that despite the very low Mn concentration (0.12 ppm) of the 127 ka coral, traces of diagenetic alteration (calcite component) were detectable by TL. Because the difference in the Mn concentration between the 127 ka coral (0.12 ppm) and the modern coral (0.15 ppm) was small, this result was not due to the increase in Mn concentration that should occur when calcite is generated by diagenetic alteration^12^. Although at present we do not have evidence, it might be effective to compare with results obtained by a microanalysis technique such as secondary ion mass spectrometry^2^. In addition, we converted the abscissa of the TL spectra shown in Fig. 4 from wavelengths to photon energy (eV) and evaluated the integrated area ratio of the calcite TL component to the aragonite (modern coral) TL component, obtaining a value of about 0.7.

It is important to explain why it is possible to detect the TL emission from very small amounts of calcite in coral skeletons. Taking the similarity of the TL mechanism between the modern and 127 ka corals into consideration, we formulated the following equation (1) (details in Supplementary information 4), which shows the time integrated TL (*A*_*TL*_) ratio of calcite to aragonite:

1 $$\frac{{A}_{TL}^{c}}{{A}_{TL}^{a}}\approx \frac{{\varphi }_{em}^{c}}{{\varphi }_{em}^{a}}\cdot \frac{{N}_{Mn}^{c}}{{N}_{Mn}^{a}}$$

where *ϕ*_*em*_, and *N*_*Mn*_ indicate the emission efficiency and the Mn concentration, respectively, and superscripts *c* and *a* refer to calcite and aragonite, respectively. As mentioned above, the integrated area ratio and the molar ratio of calcite to aragonite were approximately 0.7 and 0.025, respectively.

Here, we focused on two parameters: 1) the TL emission efficiency of Mn^2+^ and 2) the Mn partition coefficient. First, supposing that Mn is distributed according to the molar ratio of calcite and aragonite, as indicated by equation (1), the TL emission efficiency of calcite can be estimated to be about 28 times that of aragonite. The glow curves of calcite and aragonite reported by Khanlary and Townsend^32^ when a temperature of <200 °C was applied, as in this study, show that the TL emission efficiency of Mn^2+^ in calcite is clearly greater than that in aragonite; thus, our calculated result appears reasonable.

Second, with regard to the relative distribution of Mn in calcite and aragonite^12,35^, Medlin^23^ reported that the TL of natural calcite is several orders of magnitude greater than that of natural aragonite owing to the effective exclusion of manganese from aragonite, because the coordination number of Mn^2+^ is wrong for it to be readily included in the aragonite lattice. In addition, the Mn partition coefficient of calcite is more than one order of magnitude larger than that of aragonite^47^. Unfortunately, we do not have detailed information on the Mn partition coefficient in our coral samples, but it is clear that not only the TL emission efficiency but also the relative distribution of Mn in calcite and aragonite likely influence the TL properties.

The TL spectrum of the 3.5 Ma coral (Fig. 1a) showed a broad emission band centred near 620 nm; this is almost the same peak wavelength as that of the calcite component of the 127 ka coral and of the high-purity calcite sample (Fig. 4). In addition, the Mn concentration in the 3.5 Ma coral (2.0 ppm) was an order of magnitude larger than that in the modern coral (0.15 ppm). These results support that the TL spectrum of the 3.5 Ma coral includes a calcite component, though no calcite peaks were seen in the XRD spectrum. The results of a spectral separation performed using the differential data shown in Fig. 4 (calcite component) and two Gaussian functions (Fig. S3 in Supplementary information 5), though not yet sufficiently quantified, showed that a TL spectral component similar to that of the modern coral was absent, but there were emission components centred near 560 and 720 nm. Thus, in the TL spectrum of the 3.5 Ma coral, unlike in that of the 127 ka coral, a TL spectral component of the modern coral seems not to be present, presumably accounting for the large difference between the modern and the 3.5 Ma corals on the short wavelength side (Fig. 1a). However, new emission components with peaks at around 560 and 720 nm emerged. The emission component of the 560 nm peak, in particular, is similar to the CL spectra of aragonite samples^20,48^, but this emission component has hardly been reported in TL studies. The differences in the aragonite spectra between the modern and the 3.5 Ma corals may reflect a difference in the TL mechanism between them. This possibility is supported by the fact that the PL spectrum of the 3.5 Ma coral differs from the PL spectra of the modern and 127 ka corals; this difference may reflect influence of the excitation or recombination processes. A deeper understanding of the TL mechanism of the 3.5 Ma coral is needed. Then, it will be possible to estimate quantitatively the content ratio of diagenetic calcite in fossil corals, although we have no evidence that the level of calcite content influenced the oxygen isotope compositions.

**Figure 6:**
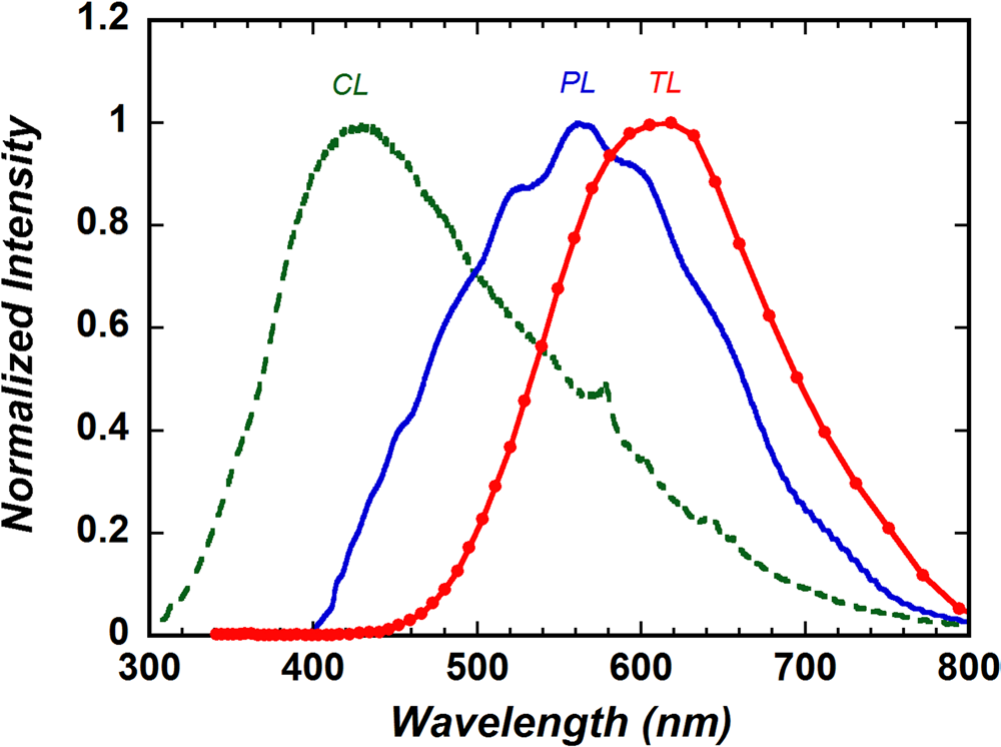
Normalized cathodoluminescence (CL), photoluminescence (PL), and thermoluminescence (TL) spectra acquired from the γ-ray-irradiated 3.5 Ma coral.

Finally, we compared the CL, PL, and TL spectra acquired from the 3.5 Ma coral after γ-ray irradiation (Fig. 6). Emission components related to Mn^2+^ (calcite, 610–620 nm; aragonite, ~560 nm; dolomite, ~660 nm)^48–50^ were not observed in the CL spectrum, which showed a relatively strong, broad emission band centred near 420 nm; this band may be due to a self-trapped exciton^51^. Additionally, as described above, the PL emission spectrum can be attributed mainly to lattice defects. Therefore, emissions from Mn^2+^ could be observed only in the TL spectrum. Thus, TL spectroscopy is a high-contrast technique that allows emission transitions from trace elements such as manganese to be observed.

## Conclusions

Using a high-sensitivity FTS, we observed the TL spectra of modern and fossil corals with Mn concentrations of approximately 0.1 ppm. The TL spectra were clearly different from the PL spectra; this difference is attributed to a difference in emission species (Mn^2+^ centre for TL and lattice defects for PL). Considering the TL mechanism, we extracted the spectral component of diagenesis-generated calcite from the TL spectra of fossil corals. Despite the very low Mn concentration (<0.1 ppm) in the diagenesis-generated calcite, the TL spectrum of calcite could be observed because the TL emission efficiency and the Mn partition coefficient in calcite are greater than those in aragonite. The TL spectroscopic technique described in this study is a potentially useful new technique for analysing diagenetic alteration and trace elements in corals.

## Electronic supplementary material

Supplementary Information

## References

[1] McGregor HV, Gagan MK (2003). Diagenesis and geochemistry of *Porites* corals from Papua New Guinea: Implications for paleoclimate reconstruction. Geochim. Cosmochim. Acta.

[2] Sayani HR (2011). Effect of diagenesis on paleoclimate reconstructions from modern and young fossil corals. Geochim. Cosmochim. Acta.

[3] Frankowiak K, Mazur M, Gothmann AM, Stolarski J (2013). Diagenetic alteration of Triassic coral from the aragonite Konservat-Lagerstätte in Alakir Çay, Turkey: Implications for geochemical measurements. Palaios.

[4] Webb GE, Price GJ, Nothdurft LD, Deer L, Rintoul L (2007). Cryptic meteoric diagenesis in freshwater bivalves: Implications for radiocarbon dating. Geology.

[5] Cuif J-P, Dauphin Y, Gautret P (1999). Composition diversity of soluble mineralizing matrices in some recent coral skeletons compared to fine-scale growth structures of fibres: discussion of consequences for biomineralization and diagenesis. Int. J. Earth Sci..

[6] Chiu T-C, Fairbanks RG, Mortlock RA, Bloom AL (2005). Extending the radiocarbon calibration beyond 26,000 years before present using fossil corals. Quat. Sci. Rev..

[7] Yokoyama Y, Esat TM (2006). Comment on “Extending the radiocarbon calibration beyond 26,000 years before present using fossil corals” by T.-C. Chiu, R.G. Fairbanks, R.A. Mortlock, A.L. Bloom (Quat. Sci. Rev. 24(2005), 1797–1808). Quat. Sci. Rev..

[8] Allison N, Finch AA, Webster JM, Clague DA (2007). Palaeoenvironmental records from fossil corals: The effects of submarine diagenesis on temperature and climate estimates. Geochim. Cosmochim. Acta.

[9] Bamston AG, Chelliah M, Goldenberg SB (1997). Documentation of a highly ENSO‐related SST region in the equatorial Pacific: Research note. Atmosphere-Ocean.

[10] Pingitore NE (1978). The behavior of Zn^2+^ during carbonate diagenesis: Theory and applications. J. Sediment. Petrol..

[11] Brand U, Veizer J (1980). Chemical diagenesis of a multicomponent carbonate system―1:Trace elements. J. Sediment. Petrol..

[12] Shen GT (1991). Paleochemistry of manganese in corals from the Galapagos Islands. Coral Reefs.

[13] Inoue M (2014). Evaluation of Mn and Fe in coral skeletons (*Porites* spp.) as proxies for sediment loading and reconstruction of 50 yrs of land use on Ishigaki Island, Japan. Coral Reefs.

[14] Moyer RP, Grottoli AG, Olesik JW (2012). A multiproxy record of terrestrial inputs to the coastal ocean using minor and trace elements (Ba/Ca, Mn/Ca, Y/Ca) and carbon isotopes (δ^13^C, Δ^14^C) in a nearshore coral from Puerto Rico. Paleoceanogr..

[15] Medlin WL (1963). Emission centers in thermoluminescent calcite, dolomite, magnesite, aragonite, and anhydrite. J. Opt. Soc. Am..

[16] Down JS, Flower R, Strain JA, Townsend PD (1985). Thermoluminescence emission spectra of calcite and Iceland spar. Nucl. Tracks..

[17] Silletti DK (2012). Radiation-induced cathodoluminescent signatures in calcite. Radiat. Meas..

[18] Waychunas GA (2014). Luminescence spectroscopy. Rev. Mineral Geochem..

[19] Calderon T (1996). Crystal field effects on the thermoluminescence of manganese in carbonate lattices. Radiat. Meas..

[20] Götte T, Richter DK (2009). Quantitative aspects of Mn-activated cathodoluminescence of natural and synthetic aragonite. Sedimentol..

[21] Orgel LE (1955). Spectra of transition-metal complexes. J. Chem. Phys..

[22] Lee YJ, Reeder RJ, Wenskus RW, Elzinga EJ (2002). Structural relaxation in the MnCO_3_-CaCO_3_ solid solution: a Mn K-edge EXAFS study. Phys. Chem. Miner..

[23] Medlin WL (1961). Thermoluminescence in aragonite and magnesite. J. Phys. Chem..

[24] Tsukino K, Satoh T, Ishii H, Nakata M (2008). Development of a multichannel Fourier-transform spectrometer to measure weak chemiluminescence: Application to the emission of singlet-oxygen dimol in the decomposition of hydrogen peroxide with gallic acid and K_3_[Fe(CN)_6_]. Chem. Phys. Lett..

[25] Suzuki A (2003). Skeletal isotope microprofiles of growth perturbations in *Porites* corals during the 1997–1998 mass bleaching event. Coral Reefs.

[26] Suzuki A (2001). Last interglacial coral record of enhanced insolation seasonality and seawater ^18^O enrichment in the Ryukyu Islands, northwest Pacific. Geophys. Res. Lett..

[27] Watanabe T (2011). Permanent El Niño during the Pliocene warm period not supported by coral evidence. Nature.

[28] Gagan MK (1998). Temperature and surface-ocean water balance of the mid-Holocene tropical western. Pacific. Science.

[29] Yang B, Luff BJ, Townsend PD (1993). Comparison between thermoluminescence and cathodoluminescence spectra of KBr and KCl. Phys. Rev. B.

[30] Van den Eeckhout K, Bos AJJ, Poelman D, Smet PF (2013). Revealing trap depth distributions in persistent phosphors. Phys. Rev. B.

[31] de Lima JF, Valerio MEG, Okuno E (2001). Thermally assisted tunneling: An alternative model for the thermoluminescence process in calcite. Phys. Rev. B.

[32] Khanlary MR, Townsend PD (1991). TL spectra of single crystal and crushed calcite. Nucl. Tracks Radiat. Meas..

[33] Kirsh Y, Townsend PD, Shoval S (1987). Local transition and charge transport in thermoluminescence of calcite. Nucl. Tracks Radiat. Meas..

[34] Townsend PD, Luff BJ, Wood RA (1994). Mn^2+^ transitions in the TL emission spectra of calcite. Radiat. Meas..

[35] Gothmann AM (2015). Fossil corals as an archive of secular variations in seawater chemistry since the Mesozoic. Geochim. Cosmochim. Acta.

[36] Parker JE (2010). A study of the aragonite-calcite transformation using Raman spectroscopy, synchrotron powder diffraction and scanning electron microscopy. CrystEngComm.

[37] Götze J, Kempa U (2008). A comparison of optical microscope- and scanning electron microscope-based cathodoluminescence (CL) imaging and spectroscopy applied to geosciences. Mineral. Mag..

[38] Rammo I, Sgi K (2006). Photoluminescence of calcite crystals of different origins. J. Appl. Spectros..

[39] Marshall SA, Reinberg AR, Serway RA, Hodges JA (1964). Electron spin resonance absorption spectrum of CO_2_^-^ molecule-ions in single crystal calcite. Mol. Phys..

[40] Wencka M, Lijewski S, Hoffmann SK (2008). Dynamics of CO_2_^−^ radiation defects in natural calcite studied by ESR, electron spin echo and electron spin relaxation. J. Phys.: Condens. Matter.

[41] Marshall SA, McMillan JA, Serway RA (1968). Electron spin resonance absorption spectrum of Y^3+^-stabilized CO_3_^3−^ molecule-ion in single-crystal calcite. J. Chem. Phys..

[42] Low W, Zeira S (1972). ESR spectra of Mn^2+^ in heat-treated aragonite. Am. Mineral..

[43] Ikeya, M. New applications of electron spin resonance: Dating, dosimetry, and microscopy. World Scientific Publishing, Singapore (1993).

[44] Barabas M (1992). The nature of the paramagnetic centres at g = 2.0057 and g = 2.0031 in marine carbonates. Nucl. Tracks Radiat. Meas..

[45] Engin B, Guven O, Koksal F (1999). Thermoluminescence and electron spin resonance properties of some travertines from Turkey. Appl. Radiat. Isot..

[46] Kontoyannis CG, Vagenas NV (2000). Calcium carbonate phase analysis using XRD and FT-Raman spectroscopy. Analyst.

[47] Kumagai T (1978). Coprecipitation of Manganese with Calcium Carbonate. Bull. Inst. Chem. Res..

[48] Richter DK, Götte T, Neuser RD (2003). Progress in application of cathodoluminescence (CL) in sedimentary petrology. Miner. Petrol..

[49] Habermann D, Neuser RD, Richter DK (1998). Low limit of Mn^2+^-activated cathodoluminescence of calcite: state of the art. Sediment. Geol..

[50] Götte T, Richter DK (2004). Quantitative high-resolution cathodoluminescence spectroscopy of smithonite. Mineral. Mag..

[51] Habermann D (2002). Quantitative cathodoluminescence (CL) spectroscopy of minerals: possibilities and limitations. Miner. Petrol..

