# Supplementary material for: Thermoluminescence of coral skeletons: a high-sensitivity proxy of diagenetic alteration of aragonite
Source: Sci Rep. 2017 Dec 21;7:17969. doi: 10.1038/s41598-017-18269-y (PMC5740112; doi:10.1038/s41598-017-18269-y)
Supplement: Supplementary file 1 — Supplementary Information [file 41598_2017_18269_MOESM1_ESM.doc]

**Supplementary Information**

***Thermoluminescence of coral skeletons:***

***a high-sensitivity proxy of diagenetic alteration of aragonite***

Noriyuki Takada^1*^, Atsushi Suzuki^2^, Hiroshi Ishii^3^, Katsuyuki Hironaka^3^

and Takayuki Hironiwa^4^

^1^Research Institute for Sustainable Chemistry, National Institute of Advanced Industrial Science and Technology, Central 5, 1-1-1 Higashi, Tsukuba, Ibaraki 305-8565, Japan

^2^Geological Survey of Japan, National Institute of Advanced Industrial Science and Technology, Central 7, 1-1-1 Higashi, Tsukuba, Ibaraki 305-8567, Japan

^3^Ueshima Seisakusho Co. Ltd., 6-5-22 Yaho, Kunitachi, Tokyo 186-0011, Japan

^4^Koga Isotope Ltd., 53-6 Jinbo, Koka, Shiga 520-3404, Japan

*Corresponding author: [n-takada@aist.go.jp](mailto:n-takada@aist.go.jp)

[ List of abbreviations]

ka: kilo-annum, 1 thousand years Ma: Mega-annum, 1 million years

TL: Thermoluminescence PL: Photoluminescence

CL: Cathodoluminescence XRD: X-Ray Diffraction

ESR: Electron Spin Resonance FTS: Fourier-Transform Spectrometer

SIMS: Secondary Ion Mass Spectrometry Gy: gray (absorbed dose, unit: J/kg)

ICP-MS: Inductively Coupled Plasma-Mass Spectrometry

**1. Comparison of** methods used to determine diagenetic alteration in coral skeletons

| Analysis technology | Principle | Advantages | Disadvantages | Sensitivity | Limit of detection | Equipment needed | Sample preparation |
| --- | --- | --- | --- | --- | --- | --- | --- |
| Thermoluminescence (TL)^53^ | Emission from material heated after pre-irradiation with γ-rays etc. | High contrast and high sensitivity for Mn | Micro-observation impossible (average informa- tion) | >0.12 ppm  for Mn | < 0.1 ppm for Mn  < 1%  for calcite | Heater + high sensitive spectrometer (FTS etc.) | Pre-irradiation with γ-rays |
| Cathodoluminescence  (CL)^50, 54^ | Emission caused by electron-impact to materials | Micro-observation (<1 µm for SEM) | Difficult to evaluate excita- tion processes | >10 ppm  for Mn | ~0.7 ppm for Mn | 1) SEM + spectrometer or  2) Optical micro- scope + spectro- meter | Polished sample surface |
| Raman Spectroscopy^55, 56^ | Vibrational spectroscopic technique based on inelastic scattering or Raman scattering | Micro-observation  (1–2 µm for SEM) | Influence of fluo- rescence emission from samples | – | ~1%  for calcite | Raman spectrometer | Polished sample surface/ finely ground sample |
| Powder X-ray Diffraction (XRD)^7, 57^ | Analytical technique based on observing the scattered intensity of X-ray | General method for diagenesis screening | Impossible to distinguish primary and secondary aragonite | >3%  for calcite | 1–2%  for calcite | X-ray diffractometer | Material is ground in ethanol to ~25 µm^*e^ |
| Secondary Ion Mass Spectrometry (SIMS)^2,12, 35^ | Analytical technique of secondary ions ejected by sputtering the material surface with primary ion beam | Micro-observation (~2 µm) / high sensitivity for trace elements | Large sensitivity difference between elements / destructive method | >10 nmol/mol for Mn/Ca  >0.56 nmol/mol for Sr/Ca | – | Secondary ion mass spectrometer | Preparation of thin section and carbon coating |

53. this study

54. Gillhaus, A. et al. Quantitative high resolution cathodoluminescence spectroscopy of diagenetic and hydrothermal dolomite. *Sediment. Geol.* **140**, 191-199 (2001).

55. Sharma, S. K., et al. Raman spectroscopy of low concentration of minerals in basaltic glass analog matrix applicable to planetary exploration. 42^nd^ Lunar and Planetary Science Conference, 1250 (2011).

56. Perrin, C. et al. Aragonite-calcite speleothems: identifying original and diagenetic features. *J. Sediment. Res.* **84**, 245-269 (2014).

57. McGregor, H. V. & Abram, N. J. Images of diagenetic textures in *Porites* corals from Papua New Guinea and Indonesia. *Geochem. Geophys. Geosyst.* **9**, 1-17 (2008).

**2. Particle size distribution**


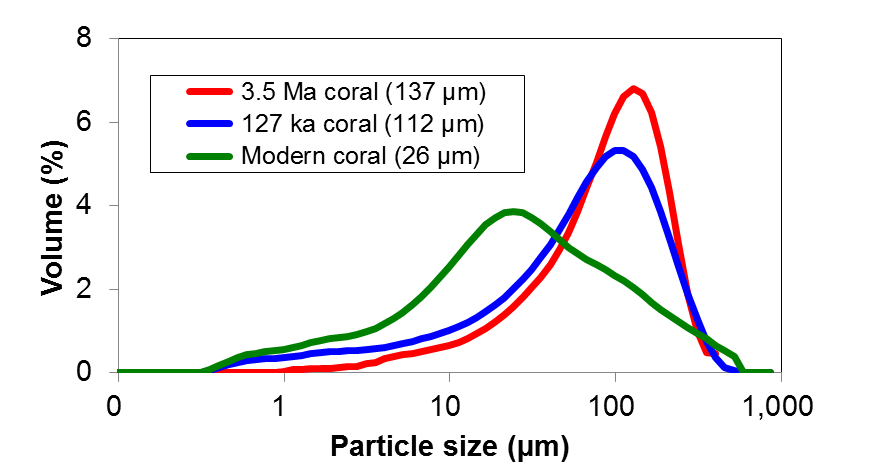


**Figure S1:** A Mastersizer 2000 with a Hydro G dispersion unit (Malvern, UK) was used to determine the particle size distribution of the modern, 127 ka, and 3.5 Ma coral samples.

**3. PL spectral changes of modern coral caused by grinding**


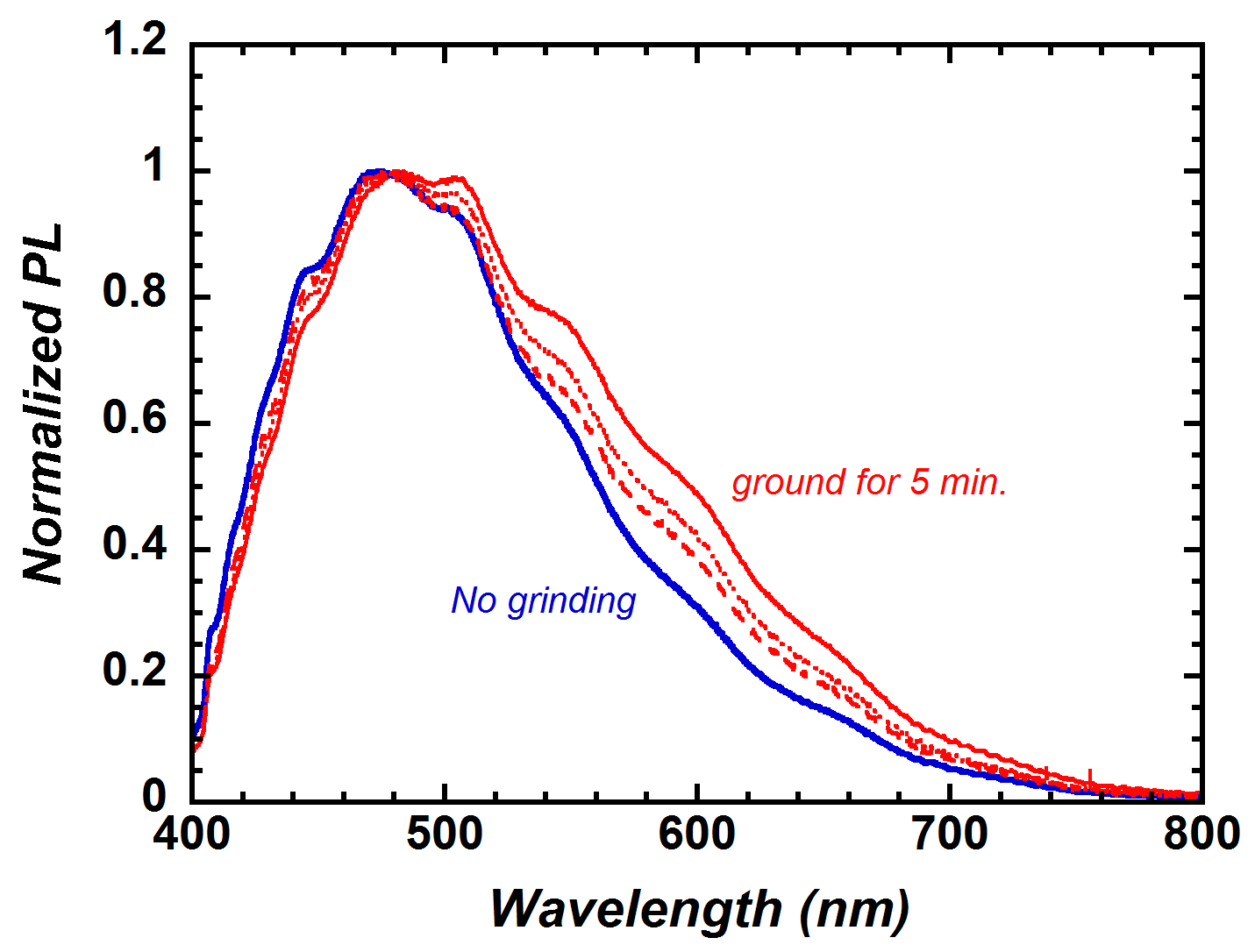


**Figure S2:** Normalized PL spectra of modern coral powder before grinding and after grinding for 5 min.

It is not easy to grind coral powders uniformly with an agate mortar and pestle; therefore, the particles observed after grinding varied from submicrometer to several tens of nanometres in size. Consequently, different PL spectra were observed depending on the observation point (Fig. S2). The PL spectra obtained after grinding were broader on the long-wavelength side than the spectrum acquired before grinding. This change is similar to that observed in the spectrum acquired after γ-ray irradiation of modern coral, as shown in Fig. 1b. In contrast, no matter how we ground the modern coral, we could not reproduce the PL spectrum of the 3.5 Ma coral. It may be necessary to perform experiments under high-temperature and high-pressure conditions.

**4. Rate equation for TL of the 127 ka coral**

Emission by Mn^2+^ is caused by the recombination between electrons released from CO_2_^–^ and CO_3_^3–^ electron centres and the holes at Mn^3+^ sites. This TL mechanism is described by equation (S-1) ^45^. The excited state (Mn^2+^)* produced by the recombination promptly falls back to the ground state Mn^2+^, emitting light as it does so. Therefore, the rate-determining step is the recombination process between electron and Mn^3+^ (first term of equation (S-1)). Equation (S-3) expresses the time evolution of TL intensity (*I_t_*). As TL emission was mostly complete within 1 min of data accumulation, the time integrated TL (*A_TL_*) can be approximated by equation (S-4):

$$Mn^{3+}\to^{e^{-}} (Mn^{2+})*\to Mn^{2+}+h\nu\begin{matrix} & \cdots\begin{matrix} & (S-1) \end{matrix} \end{matrix}$$

$$\frac{dN_{Mn^{3+}}}{dt}=-rN_{Mn^{3+}}\begin{matrix} & \cdots& (S-2) \end{matrix}$$

$$I_{t}=\varphi_{em}\cdot rN_{Mn^{3+}}=\varphi_{em}\cdot rN_{Mn^{3+}}^{0}e^{-rt}\begin{matrix} & \cdots& (S-3) \end{matrix}$$

$$A_{TL}=\int_{t} I_{t}dt\sim\varphi_{em}\cdot N_{Mn^{3+}}^{0}\begin{matrix} & \cdots& (S-4) \end{matrix}$$

where *hν*, *r*, *φ_em_*, and *N_Mn3+_* indicate TL emission, the recombination rate, emission efficiency, and the Mn^3+^ concentration (where *N^0^_Mn3+_* is the initial concentration), respectively. In equation (S-4), under the assumption that the majority of Mn in corals has the form Mn^3+^ after γ-ray irradiation ^31^, *N^0^_Mn3+_* can be replaced with the total Mn concentration *N_Mn_*.

**5. Spectral separation of the TL spectrum of the 3.5 Ma coral**


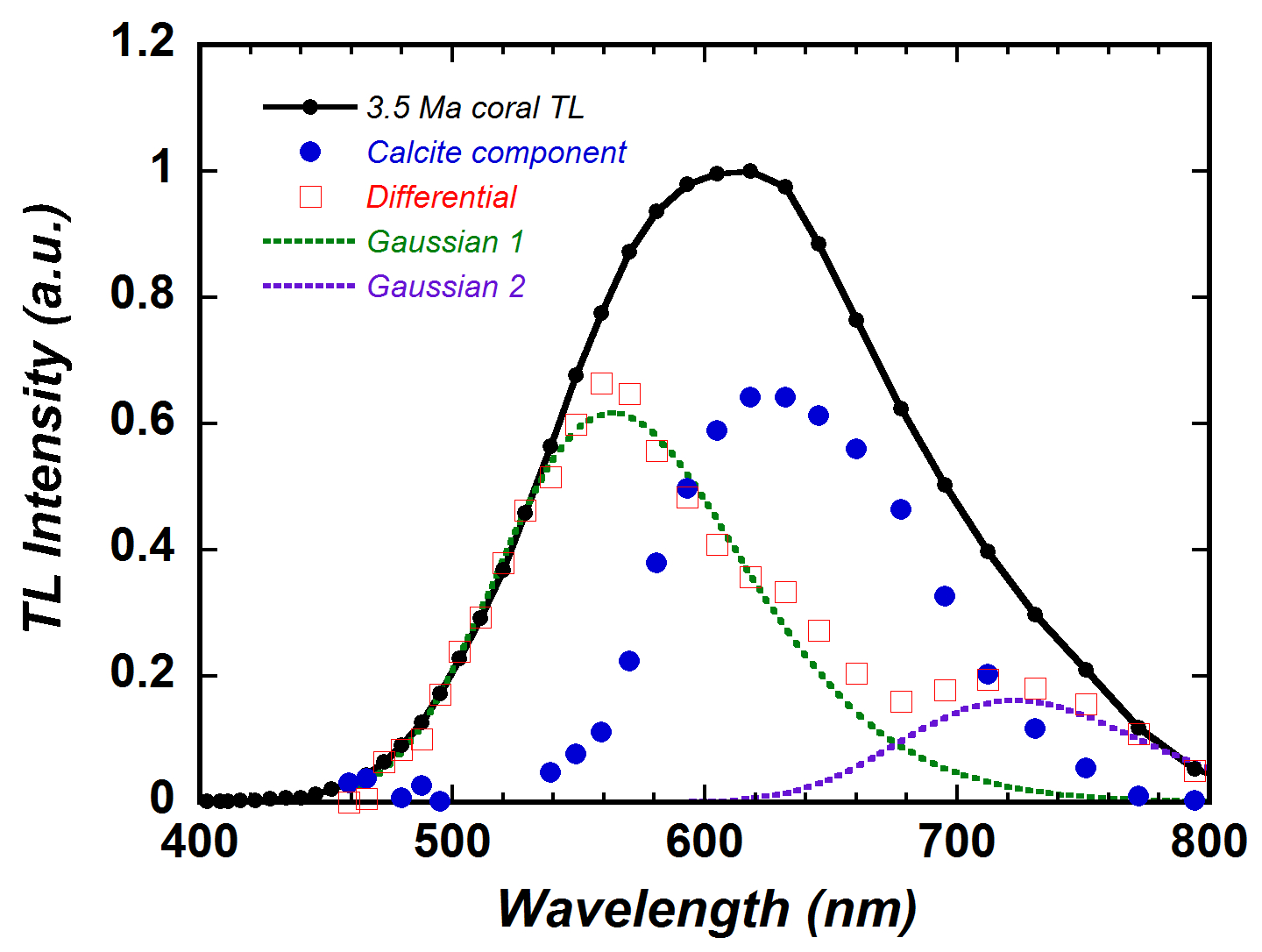


**Figure S3:** TL spectrum of the 3.5 Ma coral and the results of a spectral separation performed by using the calcite component (the differential data shown in Fig. 4) and two Gaussian functions.

The red open squares show the results after the calcite component (differential data; Fig. 4) is subtracted from the TL spectrum of the 3.5 Ma coral. The differential data were well reproduced by two Gaussian functions with peaks at approximately 560 and 720 nm. The extracted 560-nm component (Gaussian 1) is similar to the CL spectra of aragonite samples ^48,49^, but this emission component has hardly been reported in TL studies. The origin of the 720-nm component (Gaussian 2) is unclear.
